# The Application of Deep Learning for the Segmentation and Classification of Coronary Arteries

**DOI:** 10.3390/diagnostics13132274

**Published:** 2023-07-05

**Authors:** Şerife Kaba, Huseyin Haci, Ali Isin, Ahmet Ilhan, Cenk Conkbayir

**Affiliations:** 1 Department of Biomedical Engineering, Near East University, TRNC Mersin 10, Nicosia 99138, Turkey; serife.kaba@neu.edu.tr; 2 Department of Electrical-Electronic Engineering, Near East University, TRNC Mersin 10, Nicosia 99138, Turkey; 3 Department of Biomedical Engineering, Cyprus International University, TRNC Mersin 10, Nicosia 99138, Turkey; aisin@ciu.edu.tr; 4 Department of Computer Engineering, Near East University, TRNC Mersin 10, Nicosia 99138, Turkey; ahmet.ilhan@neu.edu.tr; 5 Department of Cardiology, Near East University, TRNC Mersin 10, Nicosia 99138, Turkey; cenkconk@hotmail.com

**Keywords:** coronary artery disease (CAD), coronary arteries, angiography, U-Net, pretrained models

## Abstract

In recent years, the prevalence of coronary artery disease (CAD) has become one of the leading causes of death around the world. Accurate stenosis detection of coronary arteries is crucial for timely treatment. Cardiologists use visual estimations when reading coronary angiography images to diagnose stenosis. As a result, they face various challenges which include high workloads, long processing times and human error. Computer-aided segmentation and classification of coronary arteries, as to whether stenosis is present or not, significantly reduces the workload of cardiologists and human errors caused by manual processes. Moreover, deep learning techniques have been shown to aid medical experts in diagnosing diseases using biomedical imaging. Thus, this study proposes the use of automatic segmentation of coronary arteries using U-Net, ResUNet-a, UNet++, models and classification using DenseNet201, EfficientNet-B0, Mobilenet-v2, ResNet101 and Xception models. In the case of segmentation, the comparative analysis of the three models has shown that U-Net achieved the highest score with a 0.8467 Dice score and 0.7454 Jaccard Index in comparison with UNet++ and ResUnet-a. Evaluation of the classification model’s performances has shown that DenseNet201 performed better than other pretrained models with 0.9000 accuracy, 0.9833 specificity, 0.9556 PPV, 0.7746 Cohen’s Kappa and 0.9694 Area Under the Curve (AUC).

## 1. Introduction

Coronary Artery Disease (CAD) is one of the most common forms of cardiovascular disease affecting the human population globally. CAD is the leading cause of death in both developed and developing countries [1]. About 30% of all deaths worldwide are due to cardiovascular disease. The disease is also considered one of the most frequent causes of death in Europe on an annual basis [2].

The coronary arteries supply blood to the heart muscles. This is an important function for the heart muscle and thus for the body. The Left Main Coronary Artery (LMCA) and the Right Coronary Artery (RCA) are the primary types of coronary arteries. The LMCA carries blood to the left part of the heart, whereas the RCA is mainly responsible for carrying blood to the right side of the heart. LMCA is composed of two major branches. The Left Anterior Descending artery (LAD) is one of the major branches of the LMCA that transports blood to the left anterior side of the heart. Another branch of the LMCA is the Circumflex Artery (Cx) which surrounds the heart muscle (myocardium). Through this artery, the outside and the back part of the heart are supplied with blood [3,4]. 

**Figure 1:**
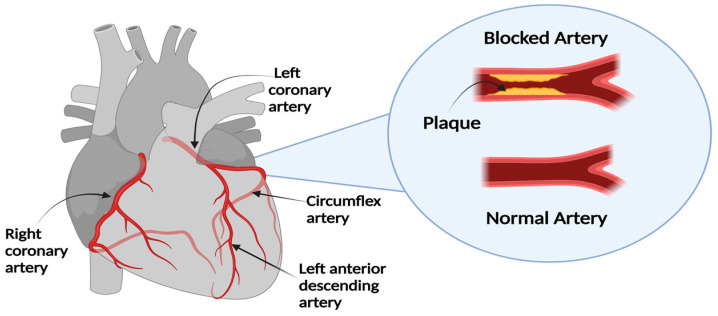
Normal versus blocked Artery.

Most heart diseases are caused by atherosclerosis, which causes the arteries to narrow or be blocked due to the build-up of plaque. CAD occurs when plaque (fatty deposits) builds up, narrowing (stenosis) the passageway and interfering with the heart’s supply of blood, oxygen, and other vital nutrients. Plaque build-up can block the coronary arteries and prevent nutrients and oxygen from reaching the heart muscle, which can lead to myocardial infraction (heart attacks) and sometimes death. When plaque is deposited on the wall of a coronary artery, the lumen of that artery is affected. Therefore, it is noteworthy that the diagnosis of stenosis can be made by measuring the lumen diameter with an angiography imaging system [2,4]. The differences between normal and blocked arteries are illustrated in Figure 1.

### 1.1. Diagnosis and Treatment of CAD

Early diagnosis of CAD significantly reduces mortality and morbidity rates [5]. In fact, for decades, CAD has been diagnosed and treated using interventional radiology procedures or surgical operations [6]. 

One of these procedures is Invasive Coronary Angiography (ICA) uses a catheter, and X-ray imaging and remains the “gold standard” for the diagnosis of coronary artery stenosis [7,8]. The primary function of the catheters used in this imaging technique is to monitor blood pressure in real-time. Blockage (occlusion) or stenosis in human arteries can be detected with the help of X-rays in which a contrast material is injected through the catheter [2]. ICA provides the cardiologist with information about the severity of luminal stenosis and is also used to diagnose atherosclerotic disease. For example, angiography can reveal calcifications, plaque ruptures severe lesions and luminal thrombosis [4].

In addition, ICA is a decision-making aid for revascularization treatments such as stent placement and coronary artery bypass surgery [9]. 

### 1.2. Challenges

Cardiologists view frames in angiographic videos with their naked eyes during angiography procedures in order to evaluate various problems affecting blood vessels, such as stenosis, plaque and blockage. Based on their experience, cardiologists can diagnose the stenosis of coronary arteries and decide medical treatment options for CAD (i.e., stent placement). However, this direct method, which requires more accuracy, objectivity, and consistency, is heavily influenced by human factors [6]. 

On the other hand, automated segmentation of the cardiovascular system has been reported to reduce the error rate in the diagnosis of stenosis. In fact, an image segmentation method has already been proposed to extract blood vessels from images so that cardiologists can quickly diagnose plaque and stenosis. 

Recently, with the development of Deep Learning (DL), various Convolutional Neural Network (CNN) architectures have been proposed and used for image segmentation [10]. CNNs are also typically used for feature extraction, image recognition, image formation, and image-based rendering. These capabilities are essential elements of the technological processes of clinical data acquisition and processing. In addition, image enhancement, image classification, and visual extraction techniques, including pattern classification, have also become very popular. The accuracy and robustness of image enhancement can also be measured using DL techniques [11]. The primary goal of using DL networks in medical image analysis is to classify images into different classes or categories (such as positive or negative, abnormal or normal, etc.). This involves analyzing the input images and classifying the output in order to determine whether or not a particular disease is present [12]. 

### 1.3. Contribution

The main research question of this study revolves around how segmentation and classification models can help increase stenosis classification performance and thus relieve the workload of cardiologists in Northern Cyprus hospitals. Consequently, the objective of this study is tailored toward applying these models to the classification of patient images. 

It is essential to mention that in this study, no artificial data were generated by data augmentation. On the contrary, all images used were clinical images and none were synthesized. Instead of developing a network from scratch, which requires a substantial amount of data, pretrained models (based on transfer learning) are used only for classification tasks, where learned parameters are transferred to solve a problem in another task. [13].

It is noteworthy to emphasize that the system proposed in this study is intended to aid cardiologists in conducting an automated and accuracy-increased diagnosis of stenosis. In the healthcare field, it is vital to minimize diagnostic errors. For this reason, decision accuracy can be significantly improved in the case that the experience and instinct of the doctors are complemented with novel automated technologies, such as Artificial Intelligence (AI). An automated diagnosis system can provide a second (additional) opinion/verification. It should be noted that using such systems on their own can result in misdiagnosis in problematic/rare cases since they do not have human-like instincts [6,11]. 

Thus, this study suggests automated diagnosis technologies be used in conjunction with health professionals.

In this study, based on the U-Net model and its variants (ResUNet-a and UNet++), we applied the DL approach to segment the major coronary arteries, including the RCA, LMCA, LAD, and Cx. Finally, five different pretrained models are used to automatically classify angiographic images with or without stenosis of the major coronary arteries. The study’s main contributions are highlighted below:

- The application of UNet, Unet++ and ResUNet-a for the automatic segmentation of coronary angiograms.
- Comparison of model’s performances for both segmentation and classification tasks.
- One of the major contributions of this study is the application of DL-based transferred learning using several pretrained models (EfficientNet-B0, DenseNet201, Mobilenet-v2, ResNet101 and Xception) for the classification of coronary angiograms.
- Comparison between the performance of models trained on the coronary artery small datasets can aid cardiologists in the selection of the best-performing model and also aid them in making appropriate decision-making.
- Another contribution of this study is the use of raw/unaltered data that are obtained from real patients by the cardiology department of Near East University Hospital, instead of using a dataset curated from an online repository system. We have noticed that a significant number of images available from online repository systems have been altered (i.e., cropped, rotated, and enlarged) to aid the segmentation and classification performance of coronary arteries. However, this is not the case in clinical applications.
- Performance evaluation of models based on accuracy, sensitivity, specificity, precision, Dice Score (F1 Score), Jaccard Index and Matthews correlation coefficient (MCC), negative predictive value (NPV), Cohen’s kappa, Area Under Curve (AUC) and Receiver Operating Characteristic (ROC) curve.

### 1.4. Related Work

This section briefly reviews previous methods relevant to our work, including conventional medical image segmentation of coronary arteries, DL-based segmentation, and image classification and diagnosis of coronary stenosis. 

Various methods have been proposed for the automatic segmentation of blood vessels based on traditional techniques. The following methods are commonly used in image processing: Filtering, thresholding, tracking-based and region growing. To illustrate, typical filtering methods utilize Gaussian and Gabor filters to extract information about edges iteratively [14]. Moreover, in tracking-based segmentation methods, seed points are selected at vessel edges, and then tracking is conducted under the guidance of constraints derived from the image. Tracking-based methods often create deformable models to capture the shape variations of coronary vessels in angiogram sequences [15].

In addition, Kerkeni et al. [16] presented a multiscale region-growing method for coronary artery segmentation. In line with this, a region-growing rule was developed that is known to uniquely integrate vessel and directional information into the region-growing approach. An iterative multiscale search is then performed based on this criterion. It is important to note that the points selected in each step serve as seed cells for the next step. 

Although these traditional image segmentation methods have improved the segmentation of vessel structures in image analysis, they could be less efficient in clinical practice due to the high computational complexity of pixel-by-pixel analysis. DL techniques using CNNs have recently gained more attention in medical imaging due to their promising results in vessel segmentation [17,18,19,20,21]. 

There are various CNN architectures that have been proposed for image segmentation. The most commonly used architectures are U-Net and SegNet models. SegNet is a DL semantic segmentation model that was originally developed for modeling scene segmentors and pixel-wise classification layers. It is an efficient architecture, especially for road and building image segmentation [22,23]. 

Zhao et al. [24] proposed a semantic segmentation algorithm to extract coronary arteries and classify them into LCA, LAD and other types of arterial segments based on the Support vector machine (SVM) algorithm using 225 angiography images. The result of the study achieved the mean accuracy of multi-class artery classification was 70.33% and the mean intersection over union for semantic segmentation of arteries was 0.6868 [24].

The U-Net model is considered the most successful architecture for medical image segmentation competitions [25]. The U-Net model is a CNN that enables efficient segmentation of biomedical data. Despite its relatively simple architecture and high accuracy in binary segmentation problems, it is also used in many other areas [26]. In addition, CNNs are used to detect specific features and to distinguish elements in the image according to their context in the original image. Several applications of these algorithms have shown promising results, including medical image processing, image enhancement, classification, and segmentation. Among the most common approaches with regard to CNNs are pretrained neural networks. This technique is conducted in order to increase the accuracy rate while significantly reducing the training time, which also increases the effectiveness of the CNN. The result of this process is what is known as transfer learning [11,27]. Among the known CNN models, GoogleLeNet is becoming increasingly popular due to its efficiency in visual object classification and recognition. The AlexNet model became the most popular after winning the ImageNet Large-Scale Visual Recognition Challenge (ILSVRC) competition in 2012, and future networks are typically compared to its achievements [28]. 

Ever since, several models have been developed and tested using ImageNet datasets. Some of the models that achieved high accuracy scores include VGGNet, Inception, ResNet, DenseNet, etc. In comparison with traditional machine learning (ML) models, DL models can automatically learn features and patterns, and classify different types of images with higher generalization. DL models have been shown to aid healthcare experts in terms of classifying medical images of diseases such as different kinds of cancers, tuberculosis and pneumonia [29].

Furthermore, data augmentation is often used to expand the range of data available to CNNs. For example, Antczak and Liberadzki [30] proposed that CNNs be used to detect stenosis. The study utilized several data augmentation techniques in order to increase the training sets using images acquired from online repositories. The developed CNN model was capable of generating random (positive and negative) stenosis patches with a Gaussian kernel. The performance evaluation of the model resulted in an overall accuracy of 90% [30]. Gil-Rios, Miguel-Angel, and colleagues proposed the use of a Support Vector Machine (SVM) for the detection of coronary stenosis from the Antczak and Liberadzki image dataset as well as the dataset of the Mexican Social Security Institute. The model performance yielded remarkable results [31]. Ovalle-Magallanes, Emmanuel, and colleagues presented a method to automatically detect coronary artery stenosis based on X-ray coronary angiograms. The images were trained using a pretrained CNN on a large amount of data, including synthetic and clinical data. The results obtained demonstrated the ability of pretrained networks in classifying coronary artery stenosis from coronary angiography images [32]. 

The study conducted by Lee et al. [33] applied deep learning for the quantification of Coronary Artery Calcium (CAC). The approach was designed according to ECG-gated coronary CT angiography (CCTA) with reference to coronary calcium scoring CT (CSCT). The data used in this retrospective study were acquired from 315 patients who underwent both CCTA and CSCT. The patients were divided into two groups, 200 in the internal and 115 in the external validation tests. The outcome of the study has shown that the automated algorithms were able to extract CACs in less than 300 s mean time with a 1.3% failure rate. The model’s volume and Agatston scores have displayed high agreement with those from CSCT with concordance correlation coefficients of 0.76–0.94 for external sets and 0.90–0.97 for external sets. In terms of the model classification performance, the study was able to record 0.94 weighted kappa and 92% accuracy for the internal sets and 0.91 weighted kappa and 86% accuracy for the external sets. 

Jamil and Roy [34] proposed the application of DL models for the detection of Valvular Heart Disease (VHD). The study employed a relatively simpler DL model in terms of network structure and performance. In order to train and validate the model performance, the study employed three different frameworks which include 1D and 2D Phonocardiography (PCG) raw signals. The frameworks revolve around the use of linear prediction cepstral coefficients (LPCC) and Mel frequency cepstral coefficients (MFCC) features for 1D PCG signal while the second framework revolves around the use several deep convolutional neural networks (D-CNNs) features for 2D PCG signals. The third framework revolves around the application of nature/bio-inspired algorithms (NIA/BIA) which include two algorithms namely, genetic algorithm (GA) and particle swarm optimization (PSO) for efficient and automatic selection of raw PCG signals. In addition, a vision transformer (ViT) was implemented in order to improve the performance of the classifiers. The evaluation of DL-based algorithms and classifiers has shown that ViT achieved the best result with an average accuracy of 99.90% and 99.95% F1-score.

Considering the fact that the manual approach for quantitatively assessing the stenosis severity of coronary arteries is very tedious and is subject to the experience of cardiologists; therefore, developing automatic quantitative coronary analysis (QCA) is crucial. In order to address this concern, Liu et al. [35] proposed the application of artificial-intelligent-based QCA also known as AI-QCA for the accurate and fast quantitative assessment of the severity of stenosis. The framework is designed according to three main units which include the boundary-aware segmentation on the coronary angiogram (CAG) images followed by the construction of the coronary artery tree which is enabled by AI and lastly the diameter fitting and detection of stenosis. The evaluation of the AI-QCA based on segmentation resulted in a precision value of 0.866, recall of 0.897 and F1-score of 0.879 using 1322 CAGs. A comparison of the diameter assessment of 249 CAGs between AI-QCA and senior experts using Root Mean Square Error (RMSE) resulted in 0.064 and 0.765 Pearson coefficients. Moreover, the use of AI-QCA has been shown to reduce the operation time from tens of minutes to a few seconds. The summary of the related work is presented in Table 1.

## 2. Methodology

**Figure 2:**
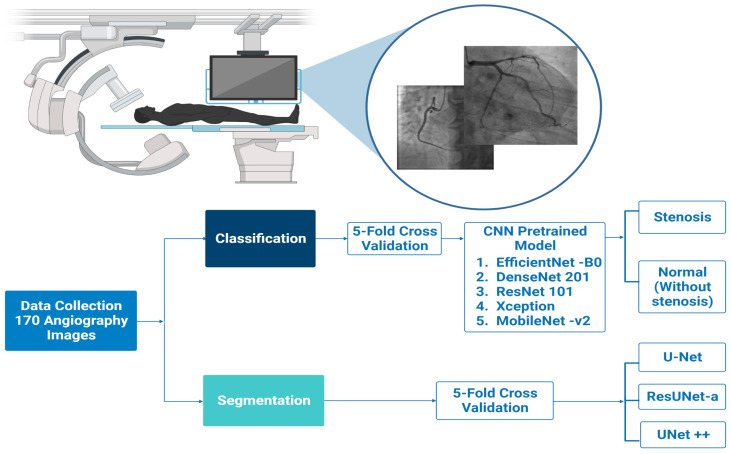
The Block Diagram of the Proposed System.

The proposed system of this study contains two main stages. The first stage consists of image pre-processing and segmentation and the second stage is comprised of image classification. The dataset is evaluated based on K-fold cross-validation set. In the first stage, U-Net, ResUNet-a, and UNet++, models are used to segment the coronary arteries. In the second stage, the coronary arteries are classified using five different pretrained models according to the presence of stenosis based on the original images of the patients. These pretrained models are evaluated using five-fold cross-validation. DenseNet201, EfficientNet-B0, Mobilenet-v2, and ResNet101 models adjust the input size of the RGB images to 224 × 224 × 3, whereas for the Xception model, the input images are resized to 229 × 229 × 3. The block diagram of the proposed system is presented in Figure 2.

### 2.1. Dataset Description

**Figure 3:**
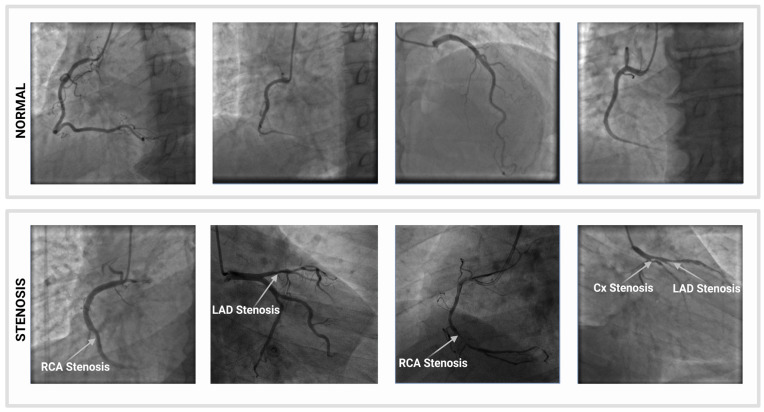
An example of images with and without stenosis in the dataset.

The dataset used in this study consisted of 170 coronary angiograms from 22 patients, including 68 narrowed (with stenosis) and 102 normal (without stenosis), acquired using the GE (General Electric) Innova 3100 Cath/Angio System (Angiography machine) at the Department of Cardiology, Near East University Hospital, Nicosia, TRNC. Image examples of coronary arteries with and without stenosis are shown in Figure 3. Patient data were collected with the approval of the hospital management ethics committee. Data collection took place between October 2020 and February 2020. Data collection was conducted according to the university’s ethical guidelines. The images captured by the medical imaging device used in this study were in Digital Imaging and Communications in Medicine (DICOM) format with a size of 512 × 512 in an RGB color model. In addition, the medical images were converted to PNG format. First, the labels for all images were created manually for segmentation by a board-certified cardiologist. Then, the catheter was marked with the major coronary arteries in the images. 

In this study, a cardiologist and an angiography technician from the Near East University Hospital analyzed the images using Syngo QCA clinical software. Catheter calibration was used as the reference standard to analyze the QCA (Quantitative coronary angiography). It should be mentioned that the QCA is one of the most commonly used tools in clinical practice to assess coronary artery stenosis. The QCA was developed to objectively measure the lumen diameter of the coronary artery [36]. 

**Figure 4:**
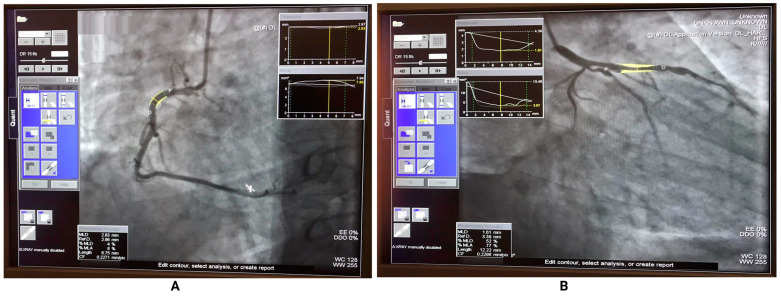
QCA analysis from our database, (**A**) An example of an arterial RCA without stenosis from our database. (**B**) An example of a LAD arterial stenosis from our database.

The scale currently proposed by the Society of Cardiovascular Computed Tomography for grading the severity of stenosis is used to rate the grade according to a standardized reporting system for patients with coronary Computed Tomography Angiography (CTA) images. The percentage of narrowing of the luminal diameter of the coronary arteries can be classified according to specific grades. According to the Coronary Artery Disease Reporting and Data System (CAD-RADS) [37], if there is “no visible stenosis,” the stenosis of the coronary arteries is classified as 0%. If the percentage of narrowing of the coronary artery is 1–24%, it is called minimal stenosis or plaque with no stenosis. It is called mild stenosis if the rate is 25–49%. If the percentage of stenosis is 50–69%, it is called “moderate”; if there is “severe stenosis,” the narrowing of the coronary arteries is categorized as 70–99%, and if the degree of narrowing (stenosis) is 100%, it is called “occluded”. The dataset used in this study was classified as coronary arteries without stenosis between 0% and 24%. Coronary arteries, with more than 24%, were classified as stenosis. Figure 4 shows an example of a coronary artery with stenosis and without stenosis from a QCA analysis performed at Near East University Hospital.

### 2.2. Image Pre-Processing

Image pre-processing was used in this study to prepare images before coronary artery segmentation. First, the images in the dataset were converted from RGB to grayscale. In addition, the images were resized to 256 × 256 to meet memory requirements by reducing training time.

### 2.3. Cross Validation

Cross-validation is a popular technique used in AI to select parameters and evaluate learning parameters and predictive performance. In this technique, the original sample is randomly divided into groups of equal size in K-fold cross-validation. Then, the training of the dataset is repeated several times for K (e.g., n = K). Finally, the average performance of the training and testing datasets is calculated as an evaluation index for the models. This approach is very effective, especially when dealing with a limited number of images, as it uses the entire dataset [38,39]. In this study, we used a five-fold cross-validation to determine training and testing sets for both segmentation and classification tasks.

### 2.4. Segmentation

#### 2.4.1. U-Net

Ronneberger et al. [40] proposed U-Net as a CNN for biomedical image segmentation. U-Net is a neural network architecture that focuses on image segmentation. There are two main paths in the U-Net architecture. The first path is considered the contracting path, also known as the encoder path or analysis path. Classification information is provided by the convolutional network, which is similar to a standard convolutional network. The second path is the expansion path, also called the decoder or synthesis path, which involves the upward convolution and concatenation of features from the contraction path. Thus, the network learns localized classification information. Furthermore, the extension path increases the output resolution, which can then be fed into a final convolutional layer to obtain a fully segmented image [41]. A convolutional network is used to create the contraction path. It consists of two 3 × 3 convolutions (unpadded convolutions) applied simultaneously, traced by a rectified linear unit (ReLU), a 2 × 2 max-pooling operation, and a stride-2 downsampling operation for each. In each downsampling stage, the number of feature channels is increased by two times their value. Each step on the expansion path is preceded by an upsampling of the feature map. The convolution path involves two 3 × 3 convolutions, followed by ReLUs, a 2 × 2 convolution (upconvolution) that halves the number of feature channels, and the concatenation of the contraction path through the corresponding feature map. Since the edge pixels are lost with each convolution, cropping is required. Convolutions of 1 × 1 are used in the last layer to divide the 64-component feature vectors into classes. There are 23 convolution layers in the network. The resulting network has a U-shaped topology and is nearly symmetric [40].

#### 2.4.2. Deep Residual U-Net (ResUNet)

The modification of U-Net gives rise to ResUNet and is categorized under the semantic segmentation neural networks. ResUnet which combines the characteristics of U-Net and the residual networks [42]. The name “ResUNet-a” is established due to the fact that the architecture is composed of residual building blocks with multiple atrous convolutions and a U-Net backbone design. ResUNet-a utilizes a U-Net encoder and decoder backbone coupled with residual connections, atrous convolutions, multi-tasking inference and pyramid scene parsing pooling which is placed in both the middle and the end of the network. The inferred tasks are applied inside the network prior to the formation of the final segmentation mask. ResUNet-a determines the boundary of the objects, the distance transform of the segmentation mask, the segmentation mask and a colored reconstruction of the input sequentially. Based on the ResUnet-a model there are two basic types of architecture, d6 and d7, which differ in depth such as the total number of layers. The encoder part of ResUNet-a d6 includes six ResBlock-a building blocks, followed by the PSPooling layer. The encoder part of ResUNet-a d7 there are seven ResBlock-a building blocks. There are three different output options for each model (d6 and d7), which are referred to as single task semantic segmentation layer, multi-task layer (mtsk), and conditioned multi-task output layer (cmtsk) [43]. 

#### 2.4.3. UNet++

U-Net++ is proposed by Zhou et al. which applied the model for the segmentation of medical images. U-Net++ is another form of the U-net model derived from the DenseNet model, using a dense network of skip connections between the expansive and the contracting paths. The architecture is known for its encoder and decoder sub-networks which are linked via a series of nested, dense skip pathways. As an updated or modified version of U-Net, U-Net++ is designed with skip pathways that link the decoder and the encoder network. Another variation between the two is that the feature map in the encoder network is straightly transmitted to the decoder network in U-Net while the feature map of the encoder network is mapped to the decoder network via dense convolution blocks through the redesigned skip pathways in U-Net++. In essence, the feature graph semantic level in the encoder is proximate to the feature graph semantic level in the decoder via dense convolution blocks [44].

### 2.5. Classification

The pretrained CNN model is already trained using millions of images (ImageNet) of different objects such as pencils, coffee mugs, cats, dogs, keyboards, etc. Most pretrained CNN models are trained on a subset of the ImageNet database, used in the ImageNet Large-Scale Visual Recognition Challenge (ILSVRC). The architecture is designed to classify up to 1000 categories [45,46].

In this study, five different pretrained CNN models were used to classify coronary angiography images. DenseNet201, EfficientNet-B0, Mobilenet-v2, ResNet101 and Xception models were used for the classification system. This section briefly summarizes these pretrained models.

#### 2.5.1. DenseNet

Increasing the model’s depth through blocks and layers has been shown to increase efficiency. DenseNet is one of these models along with ResNet with higher depths. Densely Connected Convolutional Networks (DenseNet) shared a lot of similarities with ResNet, one of the main differences between the 2 is that ResNet utilized an additive approach in which the model takes previous output as an input for future layers while DenseNet takes in all the entire previous output as an input for a future layer. DenseNet is stacked with narrow layers (i.e., 12 filters), which add a lesser set of new feature maps. Currently, there are several variants of DenseNet with the latest one as DenseNet264 followed by DenseNet201, DenseNet169 and the oldest as DenseNet121. The computational cost of this architecture is lower compared to ResNet which owes to the fact that each layer in the DenseNet model has uninterrupted access to the original input as well as a gradient from the loss function. This specific characteristic makes DenseNet one of the superior DL architectures for image classification tasks [47,48]. For example, in DenseNet201, the direct connections of the hidden layers are replaced with dense connections which enable the re-utilization of network features as well as optimize data transmission between the model’s layers. The model is designed to take in input with a size of 224 × 224 followed by a series of convolution and pooling operations. The four dense blocks of the model are interspersed with ternary transition blocks which result in an output based on 14 × 14 feature maps [49].

#### 2.5.2. EfficientNet

EfficientNet is a DL model developed by Mingxing Tan and Quoc V. Le [50]. The model provides an exceptional approach for scaling neural networks by enhancing precision, depths and widths. The CNN model’s scaling method revolves around the application of compounded coefficients that evenly scale up the resolution dimensions, width, and depth [50]. Moreover, EfficientNet is a type of DL model that is developed from baseline models established via neural network search [51]. In terms of architecture, EfficientNet-B0’s main building block is a mobile inverted bottleneck convolution (MBConv) that is slightly modified as a result of the addition of special blocks known as squeeze-and-excitation optimization blocks. Thus, each of these MBConv blocks depends on shortcut connections and depthwise convolutions between the blocks [52].

#### 2.5.3. MobileNet

MobileNet is CNN developed by a team in Google with the aim of minimizing the amount of memory used for computing and simultaneously producing high performance. The CNN architecture was trained using the large ImageNet dataset which contains thousands of categories. As the name implies, the model is designed for mobile phones or devices with lower computational power. The layers of MobileNet-v1 are divided into 2; the first layer is a depthwise convolutional layer designed for lightweight filtering (which utilized one convolutional filter for each input channel) while the second layer is a pointwise (1 × 1 convolution) layer designed for building new features through computing linear combinations of input channels. The MobileNet-v2 model is comprised of two residual blocks, the first block is designed with a stride of 1 and the second block with a stride of 2 where their main function is for downsizing. Each of these residual blocks is equipped with three main layers which include a convolutional layer (1 × 1) with a rectified linear unit (ReLU), followed by a depthwise convolution and a third of convolutional layer (1 × 1) without non-linearity. The MobileNet-v2 architecture comprises a fully convolutional layer with 32 filters followed by 19 residual bottleneck blocks [53,54].

#### 2.5.4. ResNet

ResNet is a kind of DL based on residual learning. The ResNet model was crowned the best-performing model in the 2015 ILSVRC competition. The model was developed by a team working at Microsoft. Currently, there are a handful of ResNet variants designed with a different number of layers. Some of these variants include ResNet1202, ResNet164, ResNet152, ResNet110, ResNet101, ResNet50, ResNet34 and ResNet18. Among these variants, ResNet101 is utilized in this study. There are three main layers in the ResNet101 model: convolutional layers, pooling layers, and fully connected layers. The model is designed with 101 layers with 33 residual blocks. The main function of the residual blocks of ResNet101 is to enable shortcuts or skip connections to other layers [55,56,57].

#### 2.5.5. Inception

In order to improve the training efficiency of neural networks, Google’s team first developed the Inception model back in 2014. The introduction of 1 × 1 convolutional kernels after 3 × 3 convolutional kernels, 5 × 5 convolutional kernels and 3 × 3 pooling kernels significantly minimizes the number of parameters and simultaneously increases the width of the model and optimizes the model’s adaptability to scale. The modification of the Inception model gives rise to Xception (with better performance) due to the introduction of both depthwise separable convolution and residual structure. Thus, the Xception model makes more efficient use of its parameters compared to the Inception model despite having the same number of parameters. The architecture of Xception CNN consists of 36 convolutional layers which form the feature extraction base of the CNN. In this study, the evaluation method will solely investigate image classification of CAD and thus, the convolutional base will be coupled with a logistic regression layer. Another option is to add fully connected layers before adding the logistic regression layer [58,59].

### 2.6. Experimental Design

All our experiments were conducted on a computer with 32 GB RAM, an NVIDIA GeForce RTX 2080 Ti graphics card, and an Intel i7–8th Generation CPU. We implemented all experiments with DL networks in the MATLAB 2021b programming language. For the training process, the Adam optimizer and five-fold cross-validation are employed to train and test the dataset. Coronary artery segmentation is performed using 0.0001, 100, and 8 parameters for learning rate, epoch, and batch size, respectively. The training parameters for classifying coronary arteries with and without stenosis are set to 0.0001, 100, and 32.

### 2.7. Performance Metrics and Confusion Matrix

The efficiency of the image segmentation and classification system is evaluated using a variety of performance metrics [60,61]. In this study, various performance metrics including accuracy, sensitivity, specificity, precision, Dice Score (F1 score), Jaccard Index and Matthews correlation coefficient (MCC) were used to evaluate the segmentation system.

(1) $$Accuracy=\frac{\left(TP+TN\right)}{\left(TP+FP+FN+TN\right)}$$

*Sensitivity* measures the ability of the system to segment coronary artery pixels, as seen in Equation (2).

(2) $$Sensitivity=\frac{TP}{\left(TP+FN\right)}$$

In contrast to sensitivity, *specificity* represents the segments of the background pixels and is described in Equation (3).

(3) $$Specificity=\frac{TN}{\left(TN+FP\right)}$$

*Precision* (Positive Predictive Value) represents the ratio of correctly classified coronary artery pixels to all pixels classified as coronary arteries by the system. This led the researchers to analyze the system’s success in generating true positives compared to all pixels classified as arteries, as defined in Equation (4).

(4) $$Precison\;\left(PPV\right)=\frac{TP}{\left(TP+FP\right)}$$

Image segmentation algorithms are often evaluated using the *Dice score*, which ignores the correct classification of negative samples, such as background pixels. The equation of the Dice score is given in Equation (5) [62].

*Dice score* measures the pixel similarity between the segmented image and the ground truth that ranges from 0 (no similarity) to 1 (identical) [63].

(5) $$Dice\;Score\;\left(F1\;score\right)=\frac{2TP}{\left(2TP+FP+FN\right)}$$

The *Jaccard Index* is calculated as the overlap ratio between the predicted and ground truth segmentation. This is the union area between the predicted and ground truth segmentation, as given in Equation (6) [64].

(6) $$Jaccard\;Index=\frac{TP}{\left(TP+FP+FN\right)}$$

Mathews Correlation Coefficient (*MCC*). This metric was first introduced in 1975 by a biochemist known as Brian W. Matthews who was working on the prediction of enzyme structure. As a marginal measure unaffected by the unbalanced datasets issue, the *MCC* is a contingency matrix approach to calculating the Pearson product-moment correlation coefficient between predicted and actual values. *MCC* is represented mathematically in the equation below:

(7) $$MCC=\frac{TP\times TN-FP\times FN}{\sqrt{\left(TP+FP\right)\left(TP+FN\right)\left(TN+FP\right)\left(TN+FN\right)}}$$

The interpretation of *MCC* is based on −1 as the worst value and +1 as the best value. Moreover, the metric is the only binary classification rate that produces a higher value only if the binary predictor was able to accurately predict the majority of positive and negative data cases [65].

A confusion matrix is a popular approach for estimating the performance of a model for segmentation and classification based on the number of true positives (*TP*), true negatives (*TN*), false positives (*FP*), and false negatives (*FN*) [66,67]. *TP* correctly identifies segmented coronary arteries, while *FP* identifies segmented background pixels as arteries. *TN* indicates background pixels classified as background, but *FN* incorrectly identifies arterial pixels segmented as background.

This study used accuracy, sensitivity, specificity, positive predictive value (*PPV*), and negative predictive value (*NPV*), *F*1 *score*, *MCC*, Cohen’s kappa, Area Under Curve (*AUC*) and Receiver Operating Characteristic (ROC) curve to evaluate image classification. Accuracy indicates the overall success rate of the system in classifying images with and without coronary artery stenosis. The sensitivity metric is the ability of an algorithm to detect coronary artery stenosis. Specificity refers to the power of a classification technique to see images with non-stenotic coronary arteries. The *PPV* or precision defines the probability of coronary stenosis in the images when the stenosis is present. The *NPV* indicates the probability that the result is negative when stenosis is not present. The equation of the *NPV* is given in Equation (8).

(8) $$NPV=\frac{TN}{\left(TN+FN\right)}$$

The model correctly identifies *TP*s as positive cases on images with coronary artery stenosis. *TN*s are correctly identified by the model as negative cases on images without stenosis. *FP*s occur when the model identifies coronary artery stenosis on images without stenosis according to the ground truth classification. *FN*s describe the case when the model does not identify stenosis on the image where stenosis is present according to the ground truth classification.

*The F*1 *score* is another evaluation metric that determined how precisely an ML model operates by differentiating the actual true positives from the expected ones [68]. Thus, the F1 score is regarded as the harmonic mean of recall and precision which depicts that it penalizes extreme values of both. However, one of the limitations of the *F*1 *score* is that it is not symmetric between classes as it depends solely on which class is labeled as positive or negative [69].

The kappa coefficient is another performance metric approach the is used to summarize the agreement between 2 nominal classifications according to similar categories. The evaluation metric is popularly utilized in the field of social and behavioral sciences as well as the medical field as a measure of agreement between two raters. The concept was first introduced in 1960 by Jacob Cohen which is an alternative evaluation to accuracy. The kappa coefficient is mostly used as a performance measure or sample statistic. For instance, when calculating kappa for a sample of subjects is one step in a series of experimental steps, or when kappa is used for analyzing a binary classification [65].

Cohen’s kappa can be represented mathematically [70]:

(9) $$\kappa =\frac{pA-pE}{1-pE}$$

where *p*A is the observed relative agreement between two annotators

*pE* is the hypothetical probability of agreement by chance (with data labels randomly assigned).

*κ* = 1 corresponds to the case of perfect agreement

*κ* = 0 indicates no agreement other than what would be expected by chance

AUC is regarded as one of the most significant performance metrics for evaluating the performance of ML models, especially classification tasks. *AUC*-score summarizes the Receiver Operating Characteristic (ROC) curve or is a metric that is used to represent the area under the ROC curve. The higher the score, the better the performance of the model and its ability to discriminate between positive and negative cases.

Mathematically, *AUC* is represented as: [68]

(10) $$AUC=\frac{TPR-FPR+1}{2}$$

*AUC* score is showcased as a random classification score which is represented by a diagonal ROC curve in the unit square. The value can range between 0.5 to 1, where 1 represents a perfect classification score. Nevertheless, a score range of 0.7–0.8 is acceptable while any score below 0.7 is considered as poor performance [71]. The ROC curve is graphically represented by the false-positive rate (*FPR*) and the true-positive rate (*TPR*) [51].

## 3. Experimental Results

### 3.1. Segmentation Results

**Figure 5:**
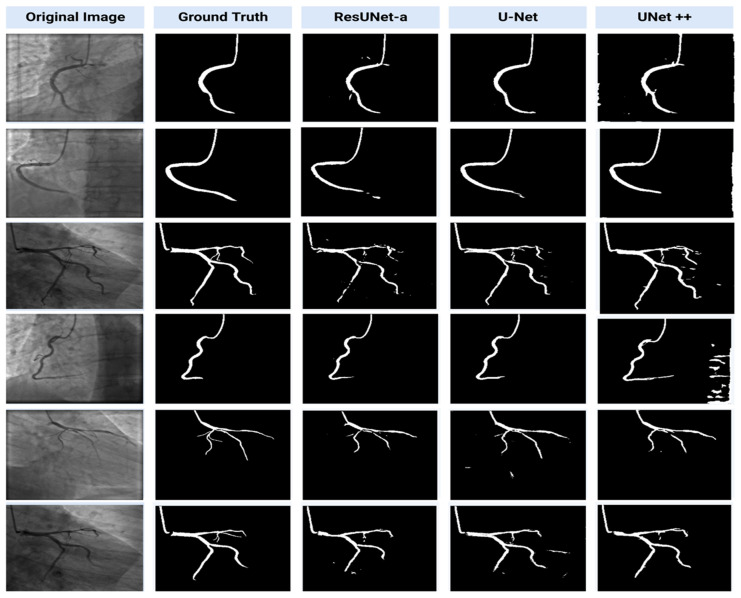
Sample segmentation results from the dataset.

The results of our segmentation study and the steps that led to these results are shown in Figure 5. The images provided include the original input image, the patient’s images marked by the cardiologist (ground truth), and the output images (segmented images) resulting from the segmentation models performed on them. The visual output results of the U-Net model are closer to ground truth images than ResUNet-a and UNet++ as seen in Figure 5.

We performed K-fold cross-validation to evaluate coronary artery segmentation and classify non-stenotic and stenotic coronary arteries using DL models. The parameter K was set to 5 in our experiments. In our dataset, for each fold, 136 images were used for the training set and 34 for the test set. All the images (total = 170) in our dataset were tested. Table 2, Table 3 and Table 4, respectively, show the K-fold cross-validation and segmentation of coronary arteries with the U-Net, ResUNet-a and UNet++ models.

Training and evaluation of U-Net yield an average accuracy of 0.9918, average Sensitivity of 0.8599, average Specificity of 0.9957, precision of 0.8423, average Dice score of 0.8467, average Jaccard Index of 0.7454 and average MCC of 0.8448 as shown in Table 2.

Training and evaluation of ResUNet-a yield an average accuracy of 0.9915, average Sensitivity of 0.8101, average Specificity of 0.9968, precision of 0.8716, average Dice score of 0.8348, average Jaccard Index of 0.7285 and average MCC of 0.8337 as shown in Table 3.

Training and evaluation of UNet++ yield an average accuracy of 0.9881, average Sensitivity of 0.8974, average Specificity of 0.9908, precision of 0.7274, average Dice score of 0.7966, average Jaccard Index of 0.6740 and average MCC of 0.7988 as shown in Table 4.

### 3.2. Classification Results

**Figure 6:**
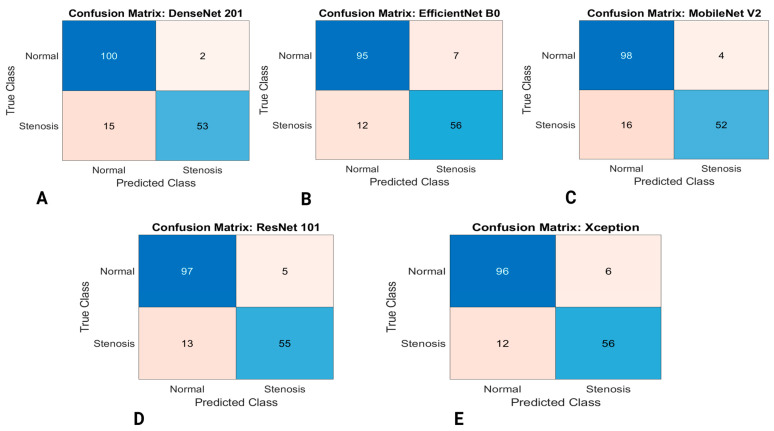
Confusion matrix for the coronary artery classification (**A**) DenseNet201 (**B**) EfficientNet-B0 (**C**) Mobilenet-v2 (**D**) ResNet101 (**E**) Xception.

The performance measures for classifying coronary arteries with and without stenosis using five different pretrained classification models with 5-fold cross-validation are presented in Table 5, Table 6, Table 7, Table 8 and Table 9. For each fold, the performance measures accuracy, sensitivity, specificity, PPV, and NPV, F1 score, MCC and Cohen’s Kappa were calculated for the system evaluation. The result of this study validated that machine learning algorithms can automatically classify coronary arteries with high accuracy. Figure 6 presents the confusion matrix of five pretrained CNN models for classifying coronary artery images as normal (without stenosis) and stenosis.

**Figure 7:**
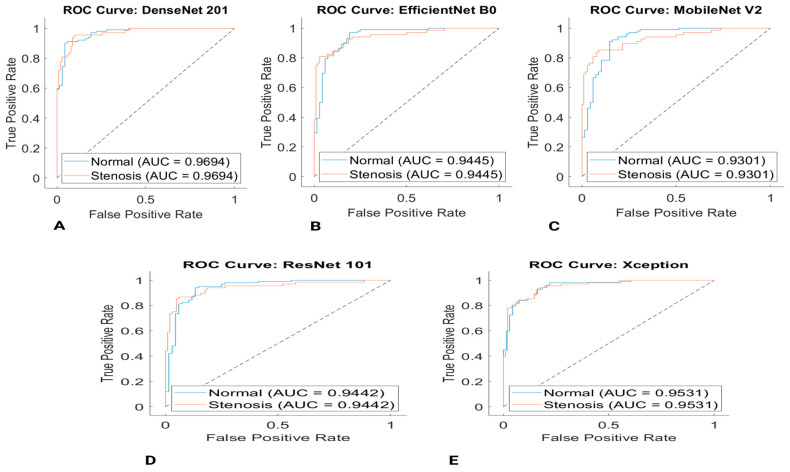
ROC curve for the coronary artery classification (**A**) DenseNet201 (**B**) EfficientNet-B0 (**C**) Mobilenet-v2 (**D**) ResNet101 (**E**) Xception.

The AUC/ROC for the classification of coronary arteries is presented in Figure 7. AUC/ROC are regarded as two of the most significant performance metrics for the evaluation of classification models. It can be seen in Figure 7. The DenseNet201 model outperformed other classification models with an AUC score of 0.9694.

Training and evaluation of the DenseNet201 model yield an average accuracy of 0.9000, average Sensitivity of 0.7699, average Specificity of 0.9833, average PPV of 0.9556, average NPV of 0.8694, average F1 score of 0.8507, average MCC of 0.7879 and average Cohen’s Kappa of 0.7746 as shown in Table 5.

Training and evaluation of the EfficientNet-B0 model yield an average accuracy of 0.8882, average Sensitivity of 0.8092, average Specificity of 0.9399, average PPV of 0.8889, average NPV of 0.8963, average F1 score of 0.8357, average MCC of 0.7655 and average Cohen’s Kappa of 0.7525 as shown in Table 6.

Training and evaluation of the MobileNet-v2 model yield an average accuracy of 0.8824, average Sensitivity of 0.7663, average Specificity of 0.9635, average PPV of 0.9233, average NPV of 0.8576, average F1 score of 0.8340, average MCC of 0.7545 and average Cohen’s Kappa of 0.7422 as shown in Table 7.

Training and evaluation of the ResNet101 model yield an average accuracy of 0.8941, average Sensitivity of 0.8021, average Specificity of 0.9540, average PPV of 0.9025, average NPV of 0.8840, average F1 score of 0.8473, average MCC of 0.7710 and average Cohen’s Kappa of 0.7652 as shown in Table 8.

Training and evaluation of the Xception model yield an average accuracy of 0.8941, average Sensitivity of 0.8206, average Specificity of 0.9472, average PPV of 0.9088, average NPV of 0.8884, average F1 score of 0.8548, average MCC of 0.7815 and average Cohen’s Kappa of 0.7711 as shown in Table 9.

## 4. Discussion

Previous studies have shown that early detection of CAD is crucial for the timely treatment and for prolonging patients’ lives. Since heart disease is the leading cause of death around the world, cardiologists are entrusted with diagnosing and treating many patients. In addition, most healthcare facilities rely on invasive coronary angiography procedures. However, despite its high accuracy and efficiency, reviewing these angiographic images and interpreting many images can be tedious for cardiologists and can also lead to misdiagnosis [2,3,4,6]. To overcome these obstacles, several studies have proposed the DL approach, in which scientists can use algorithms to classify and segment medical images [11,12,17,18,19,20,21].

In line with existing studies, this study used pretrained models to classify angiographic images. However, unlike previous studies that made use of datasets from various online repositories and many clinical datasets, this study used datasets generated by cardiologists at Near East University Hospital in North Cyprus. In addition, the classification of angiographic images (i.e., 170 images) was achieved with five pretrained CNN models as shown in Table 5, Table 6, Table 7, Table 8 and Table 9. Several studies have already used pretrained models such as DenseNet, EfficientNet and Xception to classify medical images efficiently which is in line with the result achieved [48,72,73].

### 4.1. Limitations and Clinical Implications

The growing field of AI is changing the landscape of several fields ranging from computer and electronics, medicine, agriculture, banking and finance, business, and advertisements, etc. Over the last decade, scientists have developed several platforms that integrated artificial intelligence in healthcare. Computer-aided systems are currently in use for modeling biological components (such as patient’s organs), assisted surgery (using robots), prediction of diseases outbreak, gene profiling, drug invention and repurposing and computer-aided diagnosis [74]. The use of DL models such as Artificial neural networks (ANN) and CNN are transforming the field of medical images into autonomic systems that allow real-time classification of medical data with high efficiency. This study aimed to use DL techniques for the automatic segmentation and classification of coronary arteries based on angiography images. The result achieved in this study is in line with other studies regarding the role of Computer-aided systems which receive medical data as input and produce output based on a series of algorithms. Similar approaches have been integrated into several hospitals and are currently in use to help cardiologists. Despite the fact that DL models produce excellent results, they are hindered by several challenges. For example, the lack of a limited amount of datasets can limit performance. Therefore, training DL models using a substantial amount of data is crucial for high performance. To counter this problem, scientists proposed the use of pretrained models (also known as Transfer Learning (TL) models). TL models enable scientists to extract weights and features from trained models and repurpose them on new tasks with insufficient datasets. Thus, the use of pretrained models has been shown to outperform models developed from scratch [74,75].

Another method proposed by scientists that can be used to resolve the issue of the limited amount of data is the use of data augmentation techniques which include zooming, rotation, cropping, mirroring, flipping, etc. Lastly, the last five years have seen the rise of hybrid and ensemble models. Extracting features and training using fused models have been shown to result in high performance. Moreover, the use of classifiers such as Support Vector Machine (SVM), K-nearest Neighbor (KNN) Decision Tree (DT) and Random Forrest (RF) instead of SoftMax is another dimension integrated by scientists in evaluating models with higher performances [74].

### 4.2. Comparison of Segmentation Models Performance

Based on the comparison segmentation models with the U-Net model and its variants presented in Table 10. Dice score and Jaccard index are considered more reliable and significant performance metrics for the image segmentation system [76]. Thus, a comparative assessment of the segmentation models has shown that U-Net outperformed both UNet++ and ResUnet-a in both the Dice score (0.8467) and Jaccard Index (0.7454).

### 4.3. Comparison of Classification Models Performance

Based on the comparison between different pretrained classification models as presented in Table 11, it can be seen that in terms of accuracy, the DenseNet201 model achieved the highest accuracy with 0.9000. In terms of sensitivity, Xception achieved the best result with 0.8206. While in the case of specificity, DenseNet201 achieved the best result with 0.9833. Comparative evaluation of both PPV and NPV has shown that DenseNet201 and EfficientNet-B0 achieved the best result with 0.9556 PPV and 0.8963 NPV, respectively. In the case of the F1 score, the model that achieved the highest score is Xception with 0.8548. Moreover, in the case of MCC, DenseNet201 achieved the best result with 0.7879. The comparison of the model’s Cohen’s Kappa result has shown that DenseNet201 achieved the best score with 0.7746. Therefore, in the overall assessment of performance metrics, DenseNet201 is considered the best-performing model that can automatically classify coronary arteries.

## 5. Conclusions

The need to develop accurate diagnosis approaches is crucial for minimizing diagnostic errors and increasing treatment efficiency. In line with this statement, we proposed a system that includes U-Net and its variants and pretrained CNN models for the segmentation and classification of coronary angiograms. Another contribution of this research was the use of raw data acquired from real patients instead of using a dataset collected from online repositories. This is associated with the claim that a significant number of images available in online repositories have been altered (e.g., cropped, rotated, and enlarged), to facilitate coronary artery segmentation and classification. However, this is not the case for clinical applications. This work employed U-Net, ResUNet-a, and UNet++ models for the segmentation of coronary angiography images. Consequently, we used five different pretrained models (EfficientNet-B0, DenseNet201, Mobilenet-v2, ResNet101 and Xception) to classify coronary arteries into binary classes, e.g., with or without stenosis. The main goal of this research was to implement an automatic segmentation and classification system for coronary arteries. We believe that this will help doctors make more accurate diagnoses, reduce workload, and minimize the risk of error.

It should be mentioned that one of the study’s limitations is using a small number of datasets for training and validation. In addition, the study is limited to binary classification. Therefore, future studies should focus on improving diversity in multiple coronary artery stenosis classifications according to their specific grades. Moreover, future studies will also focus on the precise measurement of coronary artery stenosis diameter using angiography images based on AI and image processing techniques. Furthermore, future studies will focus on a large number of datasets curated from various hospitals in Northern Cyprus. It is also suggested to develop an IoT/AI-based system that will allow cardiologists to upload images via online systems and obtain results in real-time.

## Figures and Tables

**Table 1:** Literature review table.

Reference	Image Acquisition	No. of Images	Method	Results
[14]	Cardiology Department of the Mexican Social Security Institute	130 coronary angiograms	Multiscale versions of the Gaussian filter and Gabor filter	ACC: 0.9698, Sens: 0.6364, Spec: 0.9880, PPV: 0.7434, Dice coefficient: 0.6857
[16]	Department of The University Hospital Fattouma Bourguiba, Monastir, Tunisia	50 coronary angiograms	Sato filter, Vessel enhancing diffusion filter, and Frangi filter Multiscale region growing	Mean precision: 82%, First dataset of Dice coefficient: 80 ± 5%, second dataset of Dice coefficient: 70 ± 5%
[18]	Asan Medical Center (Internal) Chungnam National University Hospital (External)	Internal:3302 coronary angiograms, External:181 coronary angiograms	U-Net, ResNet101, DenseNet121, InceptionResNet-v2	Average F1 score: 0.917, and 93.7% of the image
[19]	University of Michigan Hospital	462 coronary angiograms	Convolutional neural network, AngioNet	Dice score: 0.864, pixel accuracy (PA):0.983, Sens: 0.918, Spec: 0.987
[20]	Fuwai Central China Cardiovascular Hospital	109 patient’s Coronary angiogram	Threshold segmentation, Region-based segmentation, PSPNet with TL	ACC: 0.957 Sens: 0.865 Spec:0.949
[21]	Public Database	134 Coronary angiograms	CLAHE, Multiresolution strategy and multiscale strategy with U-Net	ACC: 0.9765 Sens: 0.7978 Spec: 0.9885 PPV: 0.8137 Dice coefficient: 0.7905
[31]	Mexican Social Security Institute (First dataset), Antczak and Liberadzki dataset (second dataset)	First dataset: 180 coronary angiograms (500 patches) Second dataset: 250 coronary angiograms	SVM-based classifier, UDMA	First database ACC: 0.89 and Jaccard Index: 0.80, and the second database, ACC: 0.88 Jaccard Index. 0.79
[32]	Antczak and Liberadzki dataset (public)	10,000 synthetic images and 250 real coronary angiograms images	Pretrained (VGG16, ResNet50, and Inception-v3 with Transfer Learning	ACC: 0.95, Precis: 0.93, Sens: 0.98, Spec: 0.92, and F1 score: 0.95

ACC: Accuracy, Sens: Sensitivity, Spec: Specificity, PPV: Positive predictive value, Precis: Precision, F1 score: harmonic mean of PPV (precision) and sensitivity (recall), Contrast Limited Adaptive Histogram Equalization (CLACHE), Pyramid Scene Parsing Network (PSPNet), Transfer Learning (TL), Univariate Marginal Distribution Algorithm (UMDA).

**Table 2:** The segmentation results of the K-fold cross-validation of the U-Net model (k = 5).

Folds	Accuracy	Sensitivity	Specificity	Precision	Dice Score	Jaccard Index	MCC
Fold1	0.9929	0.8477	0.9970	0.8809	0.8611	0.7628	0.8591
Fold2	0.9905	0.8355	0.9960	0.8628	0.8429	0.7390	0.8413
Fold3	0.9917	0.8946	0.9942	0.8006	0.8416	0.7446	0.8405
Fold4	0.9926	0.8397	0.9968	0.8697	0.8521	0.7542	0.8497
Fold5	0.9911	0.8822	0.9942	0.7976	0.8360	0.7266	0.8335
Average	0.9918	0.8599	0.9957	0.8423	0.8467	0.7454	0.8448

**Table 3:** The segmentation results of the K-fold cross-validation of the ResUNet-a model (k = 5).

Folds	Accuracy	Sensitivity	Specificity	Precision	Dice Score	Jaccard Index	MCC
Fold1	0.9926	0.8224	0.9975	0.8925	0.8534	0.7508	0.8518
Fold2	0.9892	0.7728	0.9966	0.8760	0.8129	0.6964	0.8134
Fold3	0.9914	0.8133	0.9960	0.8407	0.8238	0.7178	0.8211
Fold4	0.9923	0.8394	0.9966	0.8615	0.8462	0.7452	0.8445
Fold5	0.9921	0.8029	0.9974	0.8872	0.8379	0.7324	0.8376
Average	0.9915	0.8101	0.9968	0.8716	0.8348	0.7285	0.8337

**Table 4:** The segmentation results of the K-fold cross-validation of the UNet++ model (k = 5).

Folds	Accuracy	Sensitivity	Specificity	Precision	Dice Score	Jaccard Index	MCC
Fold1	0.9878	0.9128	0.9900	0.7092	0.7945	0.6673	0.7969
Fold2	0.9872	0.8673	0.9915	0.7456	0.7932	0.6637	0.7935
Fold3	0.9879	0.8937	0.9904	0.7291	0.7919	0.6779	0.7959
Fold4	0.9907	0.9046	0.9932	0.7709	0.8292	0.7176	0.8288
Fold5	0.9867	0.9086	0.9890	0.6825	0.7742	0.6435	0.7786
Average	0.9881	0.8974	0.9908	0.7274	0.7966	0.6740	0.7988

**Table 5:** The classification results of the K-fold cross-validation of the pretrained Densenet201 model (k = 5).

Folds	Accuracy	Sensitivity	Specificity	PPV	NPV	F1 Score	MCC	Cohen’s Kappa
Fold1	0.9412	0.8889	1	1	0.8889	0.9412	0.8889	0.8826
Fold2	0.8824	0.6923	1	1	0.8400	0.8182	0.7626	0.7354
Fold3	0.8529	0.7000	0.9167	0.7778	0.8800	0.7368	0.6369	0.6352
Fold4	0.9412	0.8182	1	1	0.9200	0.9000	0.8676	0.8589
Fold5	0.8824	0.7500	1	1	0.8182	0.8571	0.7834	0.7606
Average	0.9000	0.7699	0.9833	0.9556	0.8694	0.8507	0.7879	0.7746

**Table 6:** The classification results of the K-fold cross-validation of the pretrained EfficientNet-B0 model (k = 5).

Folds	Accuracy	Sensitivity	Specificity	PPV	NPV	F1 Score	MCC	Cohen’s Kappa
Fold1	1	1	1	1	1	1	1	1
Fold2	0.8529	0.6154	1	1	0.8077	0.7619	0.7050	0.6640
Fold3	0.8824	0.8000	0.9167	0.8000	0.9167	0.8000	0.7167	0.7167
Fold4	0.7941	0.8182	0.7826	0.6429	0.9000	0.7200	0.5711	0.5609
Fold5	0.9118	0.8125	1	1	0.8571	0.8966	0.8345	0.8211
Average	0.8882	0.8092	0.9399	0.8889	0.8963	0.8357	0.7655	0.7525

**Table 7:** The classification results of the K-fold cross-validation of the pretrained MobileNet-v2 model (k = 5).

Folds	Accuracy	Sensitivity	Specificity	PPV	NPV	F1 Score	MCC	Cohen’s Kappa
Fold1	0.9118	0.8333	1	1	0.8421	0.9091	0.8377	0.8247
Fold2	0.8824	0.6923	1	1	0.8400	0.8182	0.7626	0.7354
Fold3	0.8824	0.8000	0.9167	0.8000	0.9167	0.8000	0.7167	0.7167
Fold4	0.9118	0.8182	0.9565	0.9000	0.9167	0.8571	0.7954	0.7935
Fold5	0.8235	0.6875	0.9444	0.9167	0.7727	0.7857	0.6600	0.6408
Average	0.8824	0.7663	0.9635	0.9233	0.8576	0.8340	0.7545	0.7422

**Table 8:** The classification results of the K-fold cross-validation of the pretrained ResNet101 model (k = 5).

Folds	Accuracy	Sensitivity	Specificity	PPV	NPV	F1 Score	MCC	Cohen’s Kappa
Fold1	0.9706	0.9444	1	1	0.9412	0.9714	0.9428	0.9412
Fold2	0.8824	0.7692	0.9524	0.9091	0.8696	0.8333	0.7496	0.7434
Fold3	0.8529	0.7000	0.9167	0.7778	0.88000	0.7368	0.6369	0.6352
Fold4	0.9412	0.9091	0.9565	0.9091	0.9565	0.9091	0.8656	0.8656
Fold5	0.8235	0.6875	0.9444	0.9167	0.7727	0.7857	0.6600	0.6408
Average	0.8941	0.8021	0.9540	0.9025	0.8840	0.8473	0.7710	0.7652

**Table 9:** The classification results of the K-fold cross-validation of the pretrained Xception model (k = 5).

Folds	Accuracy	Sensitivity	Specificity	PPV	NPV	F1 Score	MCC	Cohen’s Kappa
Fold1	0.9412	0.8889	1	1	0.8889	0.9412	0.8889	0.8828
Fold2	0.8824	0.6923	1	1	0.8400	0.8182	0.7626	0.7354
Fold3	0.7941	0.8000	0.7917	0.6154	0.9048	0.6957	0.5548	0.5441
Fold4	0.9706	0.9091	1	1	0.9583	0.9524	0.9334	0.9312
Fold5	0.8824	0.8125	0.9444	0.9286	0.8500	0.8667	0.7677	0.7622
Average	0.8941	0.8206	0.9472	0.9088	0.8884	0.8548	0.7815	0.7711

**Table 10:** Comparison of Segmentation Models Performance of the 5-Fold Cross-Validation with the average score.

Models	Accuracy	Sensitivity	Specificity	Precision	Dice Score	Jaccard Index	MCC
U-Net	0.9918	0.8599	0.9957	0.8423	**0.8467**	**0.7454**	0.8448
ResUnet-a	0.9915	0.8101	0.9968	0.8716	0.8348	0.7285	0.8337
UNet++	0.9881	0.8974	0.9908	0.7274	0.7966	0.6740	0.7988

**Table 11:** Comparison of Classification Pretrained Models Performance of the 5-Fold Cross-Validation with the average score.

Task	Models	Accuracy	Sensitivity	Specificity	PPV	NPV	F1 Score	MCC	Cohen’s Kappa
Normal and Stenosis	Densenet201	**0.9000**	0.7699	**0.9833**	**0.9556**	0.8694	0.8507	**0.7879**	**0.7746**
	EfficientNet-B0	0.8882	0.8092	0.9399	0.8889	**0.8963**	0.8357	0.7655	0.7525
	MobileNet-v2	0.8824	0.7663	0.9635	0.9233	0.8576	0.8340	0.7545	0.7422
	ResNet101	0.8941	0.8021	0.9540	0.9025	0.8840	0.8473	0.7710	0.7652
	Xception	0.8941	**0.8206**	0.9472	0.9088	0.8884	**0.8548**	0.7815	0.7711

## Data Availability

The Institutional Review Board has not granted permission to share the patient data generated and analyzed in the current study. However, they are available on reasonable request from the corresponding author.
